# The Hospital World

**Published:** 1910-04-02

**Authors:** 


### The Hospital World.

### ROYAL BERKSHIRE HOSPITAL.

Mr. J. H. Benyon, Lord Lieutenant of Berkshire, has been elected President of the Royal Berkshire Hospital in succession to the late Major W. R. M. Thoyts. In the course of seventy years there have only been four Presidents of the hospital; Mr. Richard Benyon de Beauvoir was succeeded by Mr. Richard Benyon, and these two men held office for fifty-seven years.

### EDINBURGH INFIRMARY BOARD CHANGES.

The annual report of the Edinburgh Royal Infirmary alludes to the changes which have taken place in the composition of the board and staff. The death of the Rev. Archibald Scott, D.D., has deprived his colleagues on the board of a most highly esteemed and valued counsellor. As a clerical representative or as a representative of the contributors, he had served on the board for fourteen years, and had ungrudgingly given to the service of the Infirmary the great gifts and qualities which won him pre-eminence in other fields of service. The managers have placed on record in their minutes a tribute to the affectionate respect in which he was held by them. By the retirement of Sir James Gibson, Bart., from the board at the expiry of his office as Lord Provost of the city the managers have sustained another loss. During his tenure of the civic chair he gave a large amount of time and thought to the work of the Infirmary, and earned the gratitude of his colleagues by the interest he exhibited in its welfare. The managers regret to learn that they lose Bailie Cullen after three years' service as a member of the board. He has taken a special interest in the medical department and in a superannuation scheme (still under consideration) for the employés of the Institution. At the end of the year Lord Skerrington's term of five years' service as a representative of the contributors terminates. The managers are greatly indebted to him for the valuable assistance he has given in the administration of the infirmary, and they will greatly miss his wise counsel and unfailing courtesy. Mr. A. Scott Ireland, S.S.C., also completes his five years' term of service as representative of that Society. He has been convener of the Finance Committee for four years, and has shown conspicuous ability in that position, and proved in other respects a most useful member of the board. With deep regret the managers have also learnt that Sir Ludovic J. Grant, Bart., relinquishes office as one of those representing the University of Edinburgh on the board. He has given 13 years of valuable service to the infirmary, which the managers gratefully recognise.

### Personalia.

The resignation by Dr. Samuel English of his appointment as honorary surgeon at the Pendleton Branch Dispensary, Manchester, has been regretfully accepted.

The Earl of Dudley, the Bishop of Birmingham, the Lord Mayor of Birmingham, Sir W. Foster, Alderman Beale, Alderman Clayton, Mr. W. B. Cregoe-Colmore, and Mr. J. G. Johnson have been elected vice-presidents, and the following as members of the committee of the Birmingham and Midland Ear and Throat Hospital: Messrs. J. H. Barclay, T. W. Browett, C. S. Court, W. J. Gell, F. W. V. Mitchell, J. M. Smith, J. C. Vaudrey, and J. Whitfield.

Miss Isabel Roget has been elected a lady patroness of the Hospital for Epilepsy and Paralysis, Maida Vale, in memory of the late Mr. and Mrs. J. L. Roget, who were its generous benefactors.

Mr. T. Townrow has been placed on the board of management of the Chesterfield and North Derbyshire Hospital.

Mr. W. A. Cadbury has been re-elected President of the Birmingham and Midland Skin Hospital, to which Councillor Harris has been re-elected hon. treasurer.

Mr. L. S. Beale, Mr. J. C. Drew, Mr. Nix, Mr. H. J. Wood, and Mr. Godden have been re-elected members of the committee of the General Hospital, Tunbridge Wells.

Mr. C. E. Catling has been elected Vice-Chairman of the Hertford and Ware Joint Hospital Board.

Mr. H. Simpson Gee was elected president of the Leicester Charity Organisation Society for the ensuing year, with Rev. W. G. Whittingham as chairman of committees, Rev. Canon Sanders and Messrs. W. W. Vincent, T. B. Ellis, and A. G. Wates as vice-presidents.

Messrs. J. Magee Finny, M.D., R. O'B. Furlong, C.B., T. J. Stafford, C.B., D.L., Charles E. Jacob, J.P., Sir William Watson, D.L., and Major E. H. C. Wellesley, J.P., have been re-elected governors of the Royal National Hospital for Consumption in Ireland.

Sir James Digges La Touche, K.C.S.I., has resigned his seat on the Board of the Royal National Hospital for Consumption in Ireland owing to official duties which involved his residing in England, and the Board has co-opted Mr. Richard Caulfield Orpen.

H.R.H. the Duke of Connaught and Strathearn has been elected president of the Hampstead Hospital and Home for Incurables, the hon. secretary of which, Mr. Liddon Walters, has resigned.

Miss Evelina MacLaren, LL.B., has been elected treasurer of the Edinburgh Hospital for Women and Children in place of Mr. G. J. Evan Watson, resigned.

The Earl of Dudley has been re-elected President of the Queen's Hospital, Birmingham.

Rev. W. Hawksley has been elected chairman, Mr. Lapthorn vice-chairman, and Sir George Couzens a member of the out-patients' committee of the Royal Portsmouth Hospital.

The following gentlemen have been elected members of the executive of Nottingham Hospital Saturday Committee: Messrs. C. H. Crofts, W. Marriott, J. N. Oakland, W. H. Parrott, H. G. Pattison, and G. Taylor. Mr. George Richards was re-elected a member of the monthly board, and the following were nominated for election upon the "Special Board for the Election of Honorary Medical Officers to the General Hospital" for the ensuing year: Messrs. W. Broughton, R. B. Hallam, J. Terry, S. F. Weston, and J. Smedley. Mr. R. Reeve was elected auditor.

